# Hen Egg as an Antioxidant Food Commodity: A Review

**DOI:** 10.3390/nu7105394

**Published:** 2015-09-24

**Authors:** Chamila Nimalaratne, Jianping Wu

**Affiliations:** Department of Agricultural, Food and Nutritional Science (AFNS), 4-10 Agriculture/Forestry Centre, University of Alberta, Edmonton, AB T6G 2P5, Canada; nimalara@ualberta.ca

**Keywords:** hen eggs, naturally-occurring antioxidants, antioxidant-enriched eggs, processing, gastrointestinal digestion

## Abstract

Intake of antioxidants through diet is known to be important in reducing oxidative damage in cells and improving human health. Although eggs are known for their exceptional, nutritional quality, they are not generally considered as antioxidant foods. This review aims to establish the importance of eggs as an antioxidant food by summarizing the current knowledge on egg-derived antioxidants. Eggs have various natural occurring compounds including the proteins ovalbumin, ovotransferrin and lysozyme in egg white, as well as phosvitin, carotenoids and free aromatic amino acids in egg yolk. Some lipophilic antioxidants such as vitamin E, carotenoids, selenium, iodine and others can be transferred from feed into egg yolk to produce antioxidant-enriched eggs. The bioactivity of egg antioxidants can be affected by food processing, storage and gastrointestinal digestion. Generally thermal processing methods can promote loss of antioxidant properties in eggs due to oxidation and degradation, whereas gastrointestinal digestion enhances the antioxidant properties, due to the formation of new antioxidants (free amino acids and peptides). In summary, in addition to its well-known nutritional contribution to our diet, this review emphasizes the role of eggs as an important antioxidant food.

## 1. Antioxidants in Human

An antioxidant can be defined as “any substance that delays, prevents or removes oxidative damage to a target molecule” [1] or “any substance that directly scavenges reactive oxygen species (ROS) or indirectly acts to up-regulate antioxidant defenses or inhibit ROS production” [2]. The human body produces many enzymatic and nonenzymatic endogenous antioxidants in order to provide the primary defense against superoxide and hydrogen peroxides. The major antioxidant enzymes are superoxide dismutase (SOD), catalase (CAT), glutathione peroxidase (GPx), glutathione reductase (GRx) and peroxiredoxins [3]. Nonenzymatic endogenous antioxidants include coenzyme Q10, vitamin A, glutathione, uric acid, lipoic acid, bilirubin, l-carnitine, *etc.* [3,4]. There are many different mechanisms by which antioxidants exert protective effects against oxidative damage. They can scavenge free radicals and other reactive species by stopping initiation or propagation of free radicals chain reactions in the system, scavenging singlet oxygen, sequestering transition metal ions to prevent generation of free radicals, reducing localized oxygen concentration, and inhibiting pro-oxidative enzymes such as lipoxygenases [3,5,6]. Antioxidants can work synergistically with each other against different types of free radicals and reactive species. The most efficient enzymatic antioxidants are glutathione peroxidase (GSH-Px), catalase, and SOD [7]. GSH-Px and SOD (in two forms: CuZnSOD and MnSOD) are found in mitochondria and cytosol, whereas catalases are located in peroxisomes [8]. SOD converts superoxide into H_2_O_2_ and oxygen, while GSH-Px and catalase react with H_2_O_2_ to produce water and oxygen [8]. Although the gene expression and activity of these enzymes in the cell are well regulated to maintain redox homeostasis, internal and external factors such as aging, inflammation, smoking and toxins can influence the balance [7].

Glutathione (GSH) is a water soluble tripeptide (l-γ-glutamyl-l-cysteinylglycine) that can react with ROS using its thiol group and oxidized to form glutathione disulfide (GSSG) which can then convert back to GSH by combined action of NADPH (reduced nicotinamide-adenine di-nucleotide phosphate) cofactor and GRx [3,4]. GSH is also involved in regeneration of ascorbate [3]. Coenzyme Q10, present in all cells and membranes, is the only endogenously synthesized liposoluble antioxidant. It is an effective antioxidant which prevents lipid peroxidation during the initiation step and is involved in regenerating vitamin E [9]. Uric acid is a metabolic product of purine nucleotide, and can be absorbed back into the body during kidney filtration into the plasma [3]. A potent singlet oxygen and hydroxyl radical scavenger, uric acid prevents lysis of red blood cells by peroxidation [10].

## 2. Dietary Antioxidants

Intake of antioxidants through diet is thought to be important in reducing oxidative damage [11,12,13,14]. These antioxidants play a critical role in protecting cellular components from potentially damaging ROS and thereby maintaining homeostasis and optimal cellular functions. Synthetic antioxidants such as butylated hydroxyanisole (BHA), butylated hydroxytoluene (BHT), tert-butylhydroquinone (TBHQ) and propyl gallate (PG) have been used in both food and pharmacological applications [15]. However, because of the possible toxic and carcinogenic effects associated with BHT and BHA, their use is legally restricted [16,17]. As a result, there is a growing interest in using natural antioxidants for food and therapeutic applications which prompt the scientific community to explore new sources of natural and dietary antioxidants [3,15,18,19]. The most known groups of natural antioxidants are vitamin C, vitamin E, carotenoids and flavonoids and more recently, peptides with antioxidant properties derived from various plant and animal sources [15,19].

Most of the plant derived antioxidant compounds are phytochemicals including phenolics, flavonoids and carotenoids whereas the prominent animal-derived antioxidants are amino-derived compounds such as amino acids, peptides and proteins [20].

Vitamin E, a well-known chain breaking antioxidant, prevents propagation of lipid peroxidation reactions by donating its phenolic hydrogen to the lipid peroxyl radical [15]. Vitamin E will become a radical itself (tocopheroxyl radical), but is more stable due to delocalization of the solitary electron over the aromatic ring structure [15,21]. Lipid soluble vitamin E is considered the most important antioxidant in preventing lipid peroxidation. Carotenoids are another class of lipid soluble compounds with antioxidant properties. The main mechanisms are singlet oxygen quenching, reacting with free radicals and delocalizing the unpaired electrons with the aid of unsaturation and resonant stabilization [22,23]. Singlet oxygen scavenging ability of lutein and zeaxanthin is suggested as the main protective mechanism of eye macular against blue light-induced oxidative damage [24,25]. Carotenoids can also prevent lipid peroxidation and play a protective role in carcinogenesis [26]. Although beneficial at moderate concentration, high doses of supplementation of β-carotenoids in high concentration can act as a pro-oxidant [22,27].

Vitamin C or ascorbic acid, a water soluble vitamin, has been shown to be effective against the superoxide radical anion, H_2_O_2_, the hydroxyl radical and singlet oxygen [15,28]. It also acts synergistically with vitamin E by reacting with tocopheroxyl radical to regenerate its antioxidant ability [29]. Flavonoids represent a class of phytochemicals which are known to have antioxidant properties depending on structural features such as the number and position of the hydroxyl groups and number of phenolic rings, *etc.* [27,30]. They have been reported to scavenge peroxyl radicals, inhibit lipid peroxidation, and chelate metal ions [27,31].

Fruits, vegetables, oil seeds, nuts, cereals, spices, herbs, and grains are important sources of antioxidants such as phenolics, flavonoids and carotenoids. A great deal of research has been conducted on their antioxidant properties *in vivo*, *in vitro* as well as on extraction and purification methods, applications in food products, bioavailability, and anti-nutritional aspects [32,33,34,35,36]. Among many plant sources, berries and fruits are known for their high phenolic content including phenolic acids, and anthocyanins and their high antioxidant capacity [37]. Most vegetables including tomatoes, red pepper, *Brassica* vegetables, onion, garlic and red beet are found to have high antioxidant capacity mainly attributed to their flavonoid, carotenoid, vitamin C contents [38,39,40,41]. Although cereal grains are not considered rich sources of antioxidants compared to fruits and vegetables, grains and grain products are staple food components in the human diet and therefore their contribution is still significant [42,43,44]. The major phenolic compounds are phenolic acids such as ferulic acid, the dominating phenolic acid in wheat, caffeic acid, *p*-coumaric acid, *p*-hydroxybenzoic acid, vanillic acid and protocatechuic acid, *etc.* [44]. In addition, they contain other compounds which may exert antioxidant effects, for example, vitamin E, folates, minerals (iron, zinc) and trace elements (selenium, copper and manganese), carotenoids, *etc*. It has been suggested that antioxidant capacity of cereals is usually underestimated because of the bound phenolic compounds which do not contribute during *in vitro* assays, but can be released in the gut to exert the antioxidant activity [45,46].

Compared to antioxidants from plant sources, the available research on animal-derived antioxidants is limited. Proteins and peptides have been known to inhibit lipid oxidation through inactivation of ROS, scavenging free radicals, chelation of prooxidative transition metals, reduction of hydroperoxides, and alteration of the physical properties of food systems [47]. The most abundant antioxidant dipeptides in skeletal muscles are histidine-containing dipeptides, such as carnosine and anserine [48]. The peptide concentration varies from about 500 mg per kg of chicken thigh to 2700 mg per kg of pork shoulder depending on the type of muscle [47]. Their antioxidant properties are believed to arise through radical scavenging and metal chelation abilities [48]. The presence of thiol groups and aromatic side chains (tryptophan, tyrosine and phenyl alanine) and imidazole ring in histidine [49,50] are recognized as important structural features for their antioxidant properties. Casein derived peptides from milk proteins have been reported to inhibit enzymatic as well as non-enzymatic oxidation of lipids [51,52]. Generation of antioxidative peptides from milk proteins has been studied in detail [53]. Antioxidant peptides from egg proteins have also been reported [54,55]. Apart from proteins, other antioxidant compounds in animal tissues such as vitamin E and ascorbic acid are well-known for their antioxidant properties [56]. Some aquatic animals including salmon and shrimp contain high amounts of carotenoids with strong antioxidant properties. Astaxanthin, a carotenoid found in high concentrations in fish and shrimp, showed strong singlet oxygen and radical scavenging ability, which was 100 times greater than α-tocopherol activity [57]. The activity was mainly attributable to the presence of hydroxyl and keto endings on each ionone ring in the structure of astaxanthin [58].

## 3. Egg as an Antioxidant Food Commodity

### 3.1. Chemical and Nutritional Composition of Eggs

Egg is composed of three parts: egg shell with membranes, egg white albumen, and yolk, accounting for approximately 9.5%, 63% and 27.5% of the whole shell egg [59]. The edible portion of the egg consists of water (74%), proteins (12%), lipids (12%), carbohydrate (<1%) as well as vitamins and minerals [60]. The chemical and nutrient composition of egg is well documented [60,61,62,63]. The protein fraction is distributed in both egg white (ovalbumin, ovotransferrin, ovomucoid, ovomucin, *etc.*) and yolk (high density lipoproteins, low density lipoproteins and livetins). Eggs proteins are high quality proteins and are used as a golden standard for measuring the quality of other food proteins [61]. Almost all egg lipids are located in yolk and approximately 65% of yolk lipids are triglycerides, while phospholipids, cholesterol and carotenoids make 30%, 4%, <1%, respectively [64]. The fatty acid composition of egg yolk can be manipulated through feed formulation to produce eggs enriched with polyunsaturated fatty acids with benefits beyond basic nutrition [65]. Based on the standardized poultry feed, about 30%–35% from the total fatty acids are saturated fatty acids (SFA), 40%–45% are monounsaturated fatty acids (MUFA), and 20%–25% are polyunsaturated fatty acids (PUFA) [66]. Egg yolk lipids have been used as a source of long-chain polyunsaturated fatty acids, Docosahexaenoic acid (DHA) and phospholipids to incorporate into infant formula [67,68]. Eggs are also considered a good source of micronutrients such as vitamins and minerals. Eggs contain ~16%, 29%, 9% and 9% of the recommended daily intake (RDI) of phosphorus, selenium, iron, and zinc, and 10% of the RDI of vitamin A, D, E, K, B2, B12, biotin and pantothenic acid [61]. It has been shown that some minerals like selenium, and iodine can be enriched through fortification of feed [69,70]. In the same way as minerals, vitamin contents of egg can be manipulated through hen’s feed formulation [71].

In addition to the nutritional value, egg components have various biological activities which may render important health benefits [72]. Egg is a complete biological system designed to nourish and protect the growing embryo from various pathogen invasions. As a result, egg shell with membranes and egg white proteins possess physical and biological defense mechanisms such as viscosity, pH, antimicrobial properties, *etc*. For a list of egg compounds with various bioactivities please refer to [62] and [73].

### 3.2. Antioxidant Compounds in Eggs

Numerous compounds in both egg white and yolk exhibit antioxidant properties (Table 1). Many egg proteins such as ovalbumin, ovotransferrin, phosvitin, egg lipids such as phospholipids, as well as certain micronutrients such as vitamin E, vitamin A, selenium, and carotenoids, are reported to have antioxidant properties. In addition, eggs can be further enriched with antioxidants (*i.e.*, carotenoids, vitamin E, selenium and iodine) through manipulation of poultry feed [58,59,60,61].

**Table 1:** Antioxidants in Egg.

Name of Compound	Amount in Egg	Mechanisms of Action
*Egg white*	(% of egg white proteins)	
Ovalbumin	54	Free thiol (SH) groups in ovalbumin regulate the redox status and bind metal ions, thereby exert antioxidant properties [74]; Increased antioxidant activity when conjugated with saccharides [74,75]
Ovotransferrin	12	Possess SOD-like superoxide scavenging activity due to its metal chelating ability [76]
Ovomucin	3.5	Inhibit H_2_O_2_-induced oxidative stress in human embryonic kidney [77]
Lysozyme	3.4	Suppress reactive oxygen species (ROS) and oxidative stress genes [78]
Cystatin	0.05	Modulate the synthesis and release of NO^•^ production and thereby play a role in cellular antioxidant pathways [79,80]
*Egg yolk*	(% of yolk dry matter)	
Phosvitin	4	Antioxidant activity based on metal chelating ability; chelates iron and protects against Fe-induced oxidative damage [81,82]
Phospholipids	10	Hydrolyl amines in the side chains of phospholipids play a role in radical scavenging and exert antioxidant properties [83]
Carotenoids	<1	Unsaturated backbone and aromatic rings of carotenoids aid in neutralizing singlet oxygen and free radicals and protect against oxidative damage [22,25,26]
Vitamin E	<1	Vitamin E can donate its phenolic hydrogen to scavenge free radicals [15]; protect membrane fatty acids and plasma Low density lipoproteins (LDL), High density lipoproteins (HDL) against lipid oxidation [84]
Aromatic amino acids	<1	Aromatic nature of tryptophan and tyrosine contribute to the total antioxidant capacity [85]

#### 3.2.1. Antioxidants Naturally Occurring in Eggs

##### Ovalbumin

Ovalbumin is a glycoprotein made of 385 amino acids and constitutes approximately 54% (w/w) of the total egg white protein [60,86]. It contains six cysteine residues with a single disulfide bond and is the only egg white protein with free SH (thiol) groups [60]. The presence of thiol groups enable its ability to play a role in redox regulation and binding metal ions therefore exert antioxidant properties [87,88,89]. In 1971, Goto and Shibasaki observed the protective effects of ovalbumin against lipid oxidation in a linolenic model system [90]. When covalently attached with polysaccharides, the radical scavenging activity of ovalbumin was significantly increased [74]. It was speculated that free SH groups are responsible for the antioxidant activity of ovalbumin, which were effectively exposed upon the conjugation with polysaccharides [74]. Further studies on glycated ovalbumin showed that the activity is dependent on the type of sugars used and also the configuration of hydroxyl groups in the sugar molecule [75,91].

##### Ovotransferrin

Ovotransferrin (also known as conalbumin), representing 12%–13% of the total egg white protein, is a member of the transferrin family, a group of ion-binding proteins with an *in vivo* preference for iron [60,92]. Ovotransferrin consists of two lobes, each capable of binding one atom of Fe^3+^ and carbonate anion [60]. Among the two, the *N*-lobe is found to be more important for its antioxidant properties [76].

Ovotransferrin was reported to possess SOD-like activity against superoxide anion promoted by metal binding. The scavenging activity was dose-dependent and considerably higher than known for antioxidants such as ascorbate or serum albumin [76]. Additionally, the iron-binding ability of ovotransferrin has an indirect role in preventing iron-induced lipid peroxidation [60].

##### Lysozyme

Lysozyme is an enzyme present in almost all organisms. One egg contains approximately 0.3–0.4 g of lysozyme [93]. Lysozyme is a defensin, a member of the family of native, highly conserved host-defense proteins [78]. It contains an 18-amino acid domain that binds agents such as advanced glycation end products (AGE), which contribute to the production of ROS and increased oxidative stress (ROS). Liu *et al.*, showed that lysozyme protects transgenic mice against acute and chronic oxidative injury [78]. They also showed that hepatocytes incubated with lysozyme suppress cellular ROS levels and oxidative response genes. In another study, the survival rate following acute or chronic oxidative injury in lysozyme deficient transgenic mice was found to be significantly lower compared to the control, indicating its protective role as an antioxidant [94].

##### Cystatin

Egg white cystatin is the first identified member of the cystatin family [95]. It is a small protein of approximately 13 kDa molecular weight which makes up 0.05% of the total egg white proteins and contains two disulfide bonds [60]. Cystatin is an inhibitor of cysteine proteinases, thereby exerting antibacterial properties [96,97]. It is also reported that chicken cystatin exerts immunomodulatory activities by modulating the synthesis and release of NO^•^ production in interferon γ-activated murine macrophages [78,79,80,98,99]. Optimum levels of NO^•^ is essential for regulation of certain cellular antioxidant pathways [100]. Moreover, cystatin B, the group which chicken cystatin belongs to, has recently found to involve in protecting cerebellar granule neurons from oxidative stress by playing a role in oxidative stress-responsive signaling pathway [79]. Taken together, the role of cystatin in modulating the NO^•^ synthesis and protecting brain neurons from oxidative damage, provide us with evidence of its potential activity as an antioxidant.

##### Ovoinhibitor

Ovoinhibitor, which makes approximately 1.5% of egg white proteins, inhibits serine proteinases such as trypsin and chymotrypsin and also bacterial and fungal proteinases [93]. It was shown that chymotrypsin proteinase inhibitors including ovoinhibtor are capable of inhibiting the formation of ROS in activated human polymorphonuclear leukocytes during the inflammatory response [100]. They demonstrated that about 29% of formation of H_2_O_2_ was inhibited by ovoinhibitor at a concentration of 20 μM [100].

##### Phosvitin

Phosvitin is the most phosphorylated protein containing nearly 80% of yolk protein phosphorous and represents ~11% of yolk proteins [101]. More than half of its amino acid composition is serine, which exists as phosphoserine. It has a strong metal-binding ability and approximately 95% of yolk iron is bound to phosvitin. This high metal-binding capacity makes phosvitin a potential antioxidant, particularly against iron induced oxidative damage [82]. Iron is essential for life; under normal physiological conditions, the level is controlled by iron binding proteins ferritin and transferrin. However, if the balance is disturbed causing iron overload in cells, the effects could be lethal as humans have a very limited capacity to excrete excess iron. The excess iron in the form of Fe^2+^ can participate in Fenton reaction to produce toxic OH^•^ by reacting with H_2_O_2_.

Moreover, the circulating free iron can oxidize heart-muscle membranes, causing arrhythmia and heart failure. The iron-chelating ability of phosvitin indicates its possible role in protecting iron-induced oxidative damage. Phosvitin accelerates Fe^2+^ autoxidation, thereby reducing the availability of Fe^2+^ and inhibiting Fe^2+^-catalyzed OH^•^ generation through Fenton reaction [102]. Additionally, phosvitin is also proven to be effective against UV-induced lipid peroxidation in the presence of excess iron [103].

##### Phospholipids

Egg yolk phospholipids consist of 84% phosphatidylcholine (PC), 12% phosphatidylethanolamine (PE), 2% sphingomyelin and 2% lysophosphatidylcholine and other minor compounds [64]. King, Boyd, and Sheldon (1992) reported that egg yolk phospholipids exhibit antioxidant activity in a refined salmon oil model system, and also demonstrated that the presence of nitrogen improved the antioxidant activity of phospholipids [104]. The antioxidant activity was positively associated with the degree of fatty acid unsaturation [81]. Hydroxy amines in the side chains of choline and ethanolamines showed strong inhibition of lipid peroxidation, indicating the importance of side-chain amino acids with hydroxyl groups in the antioxidant activity [105].

##### Carotenoids

Carotenoids are lipid soluble compounds responsible for the orange-yellow color of the egg yolk. The health promoting properties of carotenoids are well documented [106,107]. More than 600 carotenoids have been identified to date and it is suggested than around 50 of them might occur in our diet and 14 in human blood [107,108]. Human body do not synthesize carotenoids and must be obtained through the diet. Therefore, it is important to consider the type and bioavailability of dietary carotenoids. Bioavailability of egg carotenoids is superior to those from green leafy vegetables [109,110] due to the solubilization of yolk lipids, which makes eggs a unique and important carrier of bioactive carotenoids. The profile of egg carotenoids is largely depend on hen’s feed composition, therefore it can vary among different types of eggs [111,112]. Certain carotenoids are allowed to use as poultry feed additives to improve color of the egg yolk, however, the amount and types of carotenoid can be varied as per the country’s feed regulation [113]. In general, lutein, zeaxanthin, canthaxanthin, β-apo-8’-carotenal, capsanthin, β-apo-8’-carotenoic acid ethyl ester, β-cryptoxanthin, and citranaxanthin can be present in egg yolk [114].

Human plasma contains several carotenoids including β-carotene, α-carotene, β-cryptoxanthin, lutein and zeaxanthin and their isomers [115]. Lutein, zeaxanthin and *meso*-zeaxanthin are the main components of the eye macular pigment [116]. Lutein and zeaxanthin are well known for their role in protecting the eye from age-related macular degeneration (AMD) [117]. The singlet oxygen and radical scavenging activity of lutein and zeaxanthin is considered one of the two major mechanisms for their beneficial effects against light-induced oxidative damage in eye macular, in particular, against AMD [117,118,119]. The other major mechanism is their ability to absorb blue light, particularly before it damages the photoreceptor cells, which is also considered a passive antioxidant action [117]. A recent study demonstrated that pre-incubation of human lens epithelial cells (HLEC) with lutein, zeaxanthin and α-tocopherol, dramatically reduced the levels of H_2_O_2_-induced protein carbonyl, MDA, and DNA damage [120]. Further, lutein, zeaxanthin and α-tocopherol supplementation increased GSH levels and GSH: GSSG ratio, particularly in response to oxidative stress [120]. Dietary supplementation with lutein reduced plasma lipid hydroperoxides and the size of aortic lesions in mice [121] and reduced the plasma levels of oxidized-LDL in guinea pigs [122], indicating a protective role in ROS induced early atherosclerosis. The ability of lutein and zeaxanthin to scavenge hydroxyl and superoxide radicals is attributed to the presence of double bonds which makes a bond with the free radical to produce a highly resonance-stabilized C-centered radical [123]. Lutein, zeaxanthin and β-cryptoxanthin have also been shown to scavenge peroxynitrite which may play a role in LDL protection against oxidative damage [124].

##### Vitamins and Minerals

On average egg contains around 1.1 mg of vitamin E [61] which is equivalent to 8.5% of RDA. Vitamin E, especially α-tocopherol as the most active form, is a well-known lipophilic chain-breaking antioxidant known to protect long-chain polyunsaturated fatty acids in the membranes of cells and thus maintain their bioactivity [125,126]. In plasma, vitamin E exists with LDL and HDL, providing protection against oxidation [84,127]. Supplementing with vitamin E increased resistance to LDL-oxidation and is associated with a lower risk of coronary diseases in both men [128] and women [129]. Eggs can be enriched with vitamin E to provide up to 150% RDA without formation of off flavour [130], not only providing the aforementioned benefits, but also protecting against oxidation of long chain fatty acids in yolk [130].

Certain minerals present in egg yolk including selenium and iodine also contribute to the antioxidant properties. Selenium is an essential mineral present in antioxidant selenoproteins such as GPx, thioredoxin reductases (TrxR) and selenoprotein P (Sepp1) [131]. Iodine has a potential role as an antioxidant in human systems including the eye, thyroid and the breast [132]. Iodine deficiency can increase the stimulation of thyroid gland by TSH resulting in excessive H_2_O_2_ [132].

##### Egg-Derived Antioxidative Peptides

Antioxidant activity was reported from egg white and egg yolk proteins. Recently, many studies have reported antioxidant properties of egg white proteins hydrolyzed using different enzymes and some have even purified the potential antioxidant peptides [133,134,135,136]. For example, Liu *et al.*, used alcalase to produce and purified three novel peptides with antioxidant properties, DHTKE (Asp-His-Thr-Lys-Glu), FFGFN (Phe-Phe-Glu-Phe-His) and MPDAHL (Met-Pro-Asp-Ala-His-Leu) [137]. Similarly, our recent studies showed that “protease P” hydrolysed egg white produce twopotent antioxidant peptides, AEERYP (Ala-Glu-Glu-Arg-Tyr-Pro) and DEDTQAMP (Asp-Glu-Asp-Thr-Gln-Ala-Met-Pro) [138]. Trypsin hydrolysate prepared from egg white protein precipitate, obtained as a by-product in cystatin and lysozyme isolation, showed a considerably better radical scavenging activity than those prepared from chymotrypsin and elastase [139,140]. Adult male spontaneously hypertensive rats fed with peptic digested egg white for 17 weeks showed increased radical-scavenging capacity of the plasma and lowered MDA concentration in the aorta, and exerted a beneficial effect on the lipid profile, lowering triglycerides and total cholesterol without changing HDL levels [141]. Two peptides derived from lecithin-free egg yolk exhibit protection against lipid peroxidation in intoxicated normal human liver cells [142]. Both peptides contained a leucine residue at their *N*-terminal positions which were thought to contribute to their antioxidant properties [142]. Another study showed that egg yolk protein hydrolysate exhibited superoxide and hydroxyl radical scavenging activity, effectively inhibiting thiobarbituric acid reactive substances (TBARS) formation from ground beef and tuna homogenates, indicating its potential as a natural antioxidant [143].

The peptide, Tyr-Ala-Glu-Glu-Arg-Tyr-Pro-Ile-Leu, derived from pepsin hydrolyzed ovalbumin, which was previously reported to possess angiotensin converting enzyme (ACE)-inhibitory activity, also exhibited a strong radical scavenging activity and delayed the LDL-oxidation induced by Cu^2+^ [54]. Peptic digests of ovalbumin inhibited the action of OH^•^ and O_2_^•−^ and also prevented the oxidation of linoleic acid in linoleic acid autoxidation system [144]. *In-vivo* studies showed that supplementation with these peptic digests of ovalbumin significantly decreased the production of oxidants and oxidative damage in serum and liver of aged mice [144].

Enzymatic hydrolysis of ovotransferrin was shown to lead to enhanced overall antioxidant activity. Two tetrapeptides (Trp-Asn-Ile-Pro and Gly-Trp-Asn-Ile) were characterized from thermolytic hydrolysate of ovotransferrin [145]. Trp-Asn-Ile was suggested as the responsible peptide motif for the high activity of the above tetrapeptides [146]. A tripeptide Ile-Arg-Trp, derived from ovotransferrin showed strong radical scavenging activity which was attributed to tryptophan and the peptide bond between Trp and Arg [146]. It is known that Trp can exert radical scavenging properties mainly due to the presence of the indole ring [147,148]. A recent study demonstrated that grafting a catechin moiety significantly increased the antioxidant activity of ovotransferrin implicating its potential as neutraceutical and functional food [149]. Peptides derived from lysozyme are reported to possess antioxidant properties [150,151,152].

Egg white ovomucin, a sulfated glycoprotein accounting for 3.5%–4% of egg white proteins, is responsible for the jelly-like structure of egg white [60,153]. Recently, ovomucin derived pentapeptide Trp-Asn-Trp-Ala-Asp was reported to reduce H_2_O_2_-induced oxidative stress in human embryonic kidney (HEK-293) cells by inhibiting intracellular ROS accumulation and blocking the ROS activated mitochondria-mediated cell apoptosis pathway [154]. Others also reported on antioxidant properties of peptides derived from ovomucin [75,134].

Phosvitin phosphopeptides (PPP) obtained from tryptic digestion of egg yolk phosvitin showed protective effects against H_2_O_2_-induced oxidative stress in human intestinal epithelial cells [155,156]. The antioxidative activity of PPP was similar to that of glutathione and positively related to the phosphorous content. PPPs are also assumed to be involved with up-regulating glutathione and associated antioxidative enzymes such as glutathione reductase, glutathione *S*-transferase, and catalase and thus reducing the oxidative stress [157]. Furthermore, the antioxidative activity of PPPs on H_2_O_2_-induced oxidative stress retained after gastrointestinal digestion [81].

#### 3.2.2. Antioxidant Enriched Eggs

Owing to its high lipid content, many lipid-soluble antioxidant compounds such as lutein/zeaxanthin, vitamin E, selenium, iodine lycopene can be incorporated into egg yolk [158]. The most studied are omega-3 fatty acids, which are incorporated into eggs by feeding fish oil, flax seed, algae, or other ingredients to laying hens [159]. High contents of omega-3 fatty acids might increase susceptibility to fatty acid oxidation therefore simultaneously enrichment of eggs with antioxidants such as vitamin E and carotenoids was suggested to decrease fatty acid oxidation and provide a good source of dietary antioxidant [158].

Carotenoids are naturally occurring in egg yolk in varied amounts depending on hen’s feed. Feed fortification with natural sources such as marigold (*Tagetes erecta*) or alfalfa (*Medicago sativa*) extracts are sources of lutein, while other sources such as corn (*Zea mays*) and red pepper (*Capsicum annuum*) provide zeaxanthin and capsanthin respectively [113,160]. Canthaxanthin, β-apo-8′-carotenal and β-apo-8′-carotenoic acid ethyl ester are chemically synthesized and incorporated into the feed [114]. Lutein and zeaxanthin are two major egg carotenoids that can be found in human serum, skin and eye macular and involved in the protective roles against oxidative stress [161,162]. Lutein content of enriched eggs can be increased up to 15-fold compared to the control group, for example enriched egg contains around 1.9 mg of lutein [130]. Lutein enriched eggs show a higher lutein bioavailability compared to lutein, lutein ester supplements, and spinach [110]. Lycopene is a hydrocarbon carotenoid reported to have strong antioxidant properties effective in reducing the risk of prostate carcinoma [163,164]. Although lycopene is not usually found in eggs, lycopene enrichment can be achieved via feed fortification with tomato powder and lycopene could reduce yolk lipid peroxidation [165].

Vitamin E is the major lipophilic antioxidant compound in our body that may provide the primary protection against free radical induced lipid peroxidation [125]. The daily requirement is approximately 15 mg α-tocopherol equivalents per day [166]. Since vitamin E is needed to protect membrane lipids from being peroxidized, this amount can be increased with higher intake of polyunsaturated fatty acids [167,168]. Egg can be enriched to provide around 20 mg of vitamin E per egg, which is more than the daily requirement, and also provide protection against unsaturated fatty acid peroxidation [158]. Folate, a water soluble B-group vitamin is shown to reduce the incidence of neural tube defects in newborns [169]. Egg yolk can be enriched with highly bioavailable folate through fortification of feed with folic acid to provide up to 12.5% of the recommended daily intake of folate [170,171]. Almost all the folate in egg exists in the form of 5-methyltetrahydrofolate (5-MTHF), and showed high stability during cooking [172]. Folates are reported to have antioxidant properties and among different forms, 5-MTHF was reported to have the most prominent antioxidant activity, which was attributed to the electron donating effect of the 5-amino group [173]. *In vivo* and *ex vivo* studies with human vessels showed that 5-MTHF improves NO-mediated endothelial function, decreases superoxide production, scavenge peroxynitrite and also reversed endothelial nitric oxide synthase (eNOS) uncoupling, thereby exerts antioxidant effects [174,175].

Both selenium and iodine, which are known to have antioxidant properties, can be effectively transferred into the egg yolk. Eggs can be supplemented to provide up to 50% and 150% of the daily requirements of selenium and iodine respectively [158,176]. Collectively, these antioxidant enriched eggs provide multiple advantages by serving as a dietary source of several nutrients including omega 3, vitamin E, vitamin D, selenium, iodine and also as an important source of antioxidants such as lutein.

### 3.3. Effect of Processing, Storage Conditions and Gastrointestinal Digestion on Egg Antioxidants

Foods are subjected to various processing and storage conditions before consumption, which may influence the antioxidant capacity of food components. The effect of food processing and storage conditions on the overall antioxidant activity of a particular food is a result of several different events occurring consecutively or simultaneously. According to Nicoli *et al.*, there are three possible effects of food processing on the overall antioxidant capacity [177]:

1. The total antioxidant capacity is not affected: as a result of no changes in natural antioxidant compounds or loss of naturally occurring antioxidants balanced by formation of compounds with novel or improved antioxidant properties,
2. The total antioxidant capacity is increased: as a result of improvement of antioxidant properties of naturally occurring compounds or formation of new antioxidant,
3. The total antioxidant capacity is decreased: loss of naturally occurring antioxidants or formation of new compounds with pro-oxidant activity.

Most thermal processing methods can create environments that can lead to oxidation, thermal degradation, and leaching of vitamin C and phenolic compounds, which would reduce the antioxidant activity [178]. With regard to carotenoids, processing can lead to the dissociation of compounds from plant matrix resulting in increased carotenoid antioxidants, and improved digestive absorption [179,180,181]. Most of the fruits and vegetables contain phenolic compounds, carotenoids and vitamin C, which are differently affected by processing conditions. Consequently, the total antioxidant capacity can be increased [182,183,184] or decreased [184,185,186,187]. In animal-derived foods, the antioxidant capacity depends mainly on amino compounds (proteins, peptides and amino acids) and vitamin E.

Heat modification of egg white proteins, ovalbumin, lysozyme and ovomucoid via Maillard reaction resulted in protein-sugar conjugates, leading to increased radical scavenging properties [188]. Chen, Chi, and Xu showed that there are no significant differences in terms of DPPH (1,1-diphenyl-2-picrylhydrazyl) radical-scavenging activity, reducing power, and lipid peroxidation inhibitory activity of spray dried and freeze-dried egg white protein hydrolysates compared to the undried sample [135]. Antioxidant properties of egg yolk phosvitin is due to its iron binding abilities; heating phosvitin at 110 °C for 40 min did not change the iron binding ability of phosvitin [189].

Carotenoids and vitamin E in egg yolk are reported to be influenced by thermal processing. In the presence of heat, light, oxygen, *etc.*, carotenoids can undergo *trans-cis* isomerization, or they can be degraded resulting in altered or loss of bioactivity [190]. Boiling of eggs resulted in a 10%–20% carotenoid loss [112], whereas pasteurizarion did not change the carotenoid content [191]. Storage conditions such as temperature can also affect the antioxidant properties of eggs. Storage at refrigeration temperature for two weeks reduced significantly the total carotenoid content in raw eggs enriched with omega-3 and carophyll (canthaxanthin preparation), while at room temperature, the losses were observed after 7 days of storage [192]. The vitamin E content of eggs was also significantly reduced by thermal processing accompanied with increased lipid oxidation products [193,194].

Pretreatments with ultrasound, high-intensity pulsed electric field (PEF) or high pressure can affect the antioxidant activity of egg proteins/peptides. Pretreatment with PEF significantly increased the antioxidant activity of egg white protein hydrolysate which was attributed to the release of free amino acids and small peptides with antioxidant properties [134]. Also, high pressure processing and sonication or ultrasound pretreatments are shown to improve the degree of hydrolysis of egg white proteins which result increased antioxidant properties [195,196].

Gastrointestinal digestion involves extreme pH conditions and various enzymes which might cause degradation of antioxidant componds or generation of novel antioxidant compounds. Many recent research activities have evaluated the changes in antioxidant capacity of different food products after gastrointestinal digestion using diverse model systems. After gastrointestinal digestion, the antioxidant activity of wheat [197], gooseberries [198], grapes [199], soymilk [200], saithe and shrimp [201] and loach protein hydrolysate [202] increased several times, attributed mainly to increased free amino acid content and short chain peptides generated during digestion. However, the antioxidant activity of some foods such as apples [203] and *Feijoada* whole meal (a traditional Brazilian dish containing vegetables) [204] was significantly reduced.

Many studies have reported the formation of antioxidant peptides after simulated gastrointestinal digestion of egg components [81,134,150]. A recent study showed that different types of domestic cooking methods such as boiling and frying decreased the antioxidant activity [134]. Nevertheless, simulated gastrointestinal digestion of cooked egg with pepsin and pancreatin significantly increased the antioxidant activity, which was attributed to the release of amino acids and antioxidant peptides [134]. Our recent studies on effect of simulated gastrointestinal digestion of egg yolk antioxidant using highly sophisticated intestinal model TIM-1 showed that, lutein and zeaxanthin, the main egg carotenoids, remain stable during the gastrointestinal digestion and also highly bioaccessible possibly due to the association with yolk fat [205], it is likely that they retain their antioxidant activity. Moreover, gastrointestinal digestion significantly increased the total antioxiant activity of cooked egg yolk (about 5–8 fold), which is presumed to be a result of increased free amino acid content and release of antioxidant peptides

## 4. Summary

Oxidative stress is hypothesized to be responsible for the onset and development of various diseases and ageing. Dietary antioxidants are thought to impart potential benefits in reducing the risk of some chronic diseases by maintaining redox homeostasis. There is extensive research on the presence and characterization of antioxidants from fruits, vegetables, cereals and herbs; however, there is only limited research with regard to antioxidants from animal products. Eggs are an important part of our breakfast and an excellent source of high quality proteins, lipids, vitamins and minerals. Many egg proteins such as ovalbumin, ovotransferrin, phosvitin, and egg lipids such as phospholipids, as well as certain micronutrients such as vitamin E, vitamin A, selenium, and carotenoids, are reported to have antioxidant properties. Furthermore, eggs can be enriched with antioxidants (*i.e.*, carotenoids, vitamin E, selenium and iodine) through manipulation of poultry feed. Domestic cooking tended to reduce the antioxidant activity of egg, while gastrointestinal digestion of cooked eggs increased the antioxidant due to the release of amino acid and peptides.
